# Supplementary material for: De novo assembly of the freshwater prawn Macrobrachium carcinus brain transcriptome for identification of potential targets for antibody development
Source: PLoS One. 2021 Apr 9;16(4):e0249801. doi: 10.1371/journal.pone.0249801 (PMC8049718; doi:10.1371/journal.pone.0249801)
Supplement: Attachment — Submitted filename: Review of PONE-D-21-00863.pdf [file pone.0249801.s003.pdf]

Review of PONE-D-21-00863
De novo assembly of the freshwater prawn *Machrobrachium carcinus* brain transcriptome for identification of potential targets to develop antibodies specific for crustacean neural structure and function markers

Authors: Crooke-Rosado et al.
Corresponding author: Maria A Sosa

Summary. This manuscript is a very important but straight-forward contribution describing the generation of a transcriptome from the brain of a prawn, and the detection of five glutamate receptor transcripts with homologies to known arthropod and mammalian sequences. The work described makes a significant contribution to our knowledge about glutamate receptors crustacean species, and alludes to the production of antibodies and localization efforts that are now underway based on the transcriptome analysis presented. I enjoyed reading this paper very much!

The presentation of this transcriptome work is clear and accessible. The motivations for pursuing this work (use of crustacean species as bioindicators, lack of transcriptomic information about the nervous system and particularly glutamate receptors) are clearly articulated in the introduction. The seven figures are thoughtfully composed to illustrate the results of their analysis, and Figures 3-7 present the glutamine receptor contig sequence data, antigenic propensity values and 3D reconstruction models. The text is exceptionally well organized, and is written using smooth and direct language. I have no major criticisms of this manuscript, but do have a few suggestions for improvement, enumerated below.

1. The title seems long and cumbersome. I suggest perhaps "*De novo* assembly of the freshwater prawn *Macrobrachium carcinus* brain transcriptome for identification of potential targets for antibody development". I think the rest of the original title is actually implicit ---if antibodies are being generated against *M. carcinus* sequences from brain, these antibodies will by definition be useful as markers in the nervous system.

2. Although glutamate is often referred to as an "excitatory neurotransmitter", this is actually a misnomer because the effect of a transmitter is defined by the receptor ---not the transmitter. As one example provided in this manuscript, the GluR4 receptor has some inhibitory effects. I suggest rewording "excitatory neurotransmitter" (found on lines 101 and 394), e.g., generally involved in excitatory influences.

3. I believe that the models in part D of Figures 3-7 actually describe the tertiary structure of these contig sequences (not secondary structure as stated in the text and legends), because folds indicative of the side chain interactions are indicated.

Minor editorial suggestions:

Line 54: constituents "of" (not to)

Line 86: American lobster ("American" should be capitalized)

Line 92: "…and pigment-dispersing hormone" ("the" is awkward here)

Lines 105-108: This sentence is long and difficult to read. How about "Feinstein and colleagues….presynaptic membranes of neuromuscular junctions in the crayfish *Procambarus clarkii*, using electron microscopy and immunocytochemistry with both monoclonal and polyclonal antibodies against the mammalian NMDA receptor."

Line 110: "specific in the thoracic appendages" (not "at")

Line 131: "continuously filtered"

Line144: "filter cartridge" does not need to be capitalized

Line 165: "related to" (rather than "with")

Line 187: "…markers in the prawn CNS and neuromuscular junction…"

Line 211: "a total…was annotated"

Line 220: "associated with" (not "to")

Line 239: "which contigs" (not "what")

Line 251: "when examined individually" (not "looked")

Line 252: "above 1, are predicted to elicit"

Line 265: I suggest that after the comment "showing 6 determinants", you might include in parenthesis "(peaks in graph)" for those who are not familiar with these plots.

Line 270: "shows reliable sequence conservation" (remove "a")

Line 341: "European" and "Indian" should be capitalized.

Line 379: use present tense here? "…this is the first study that reports genomic information"

Line 392: "on identifying" (rather than "in")

Line 397: "…about glutamate receptor distribution and localization…" (possessive not necessary)

Line 406: "*Xenopus laevis*" ??

Line 430: "…and their most potent agonist…" (should be plural, "their")

Line 438: This needs clarification, as it's not clear what a "mammalian-raised" antibody is. I believe you are trying to say "…using an antibody raised against the mammalian form of the receptor…"

Line 440: "This provides the advantage…"

Line 443: "increasing the reproducibility of labeling."

Line 444: "With the transcriptome contribution and the availability…"
