# Supplementary material for: De novo assembly of the freshwater prawn Macrobrachium carcinus brain transcriptome for identification of potential targets for antibody development
Source: PLoS One. 2021 Apr 9;16(4):e0249801. doi: 10.1371/journal.pone.0249801 (PMC8049718; doi:10.1371/journal.pone.0249801)
Supplement: Attachment — Submitted filename: Response to Reviewers - FINAL.pdf [file pone.0249801.s004.pdf]

UNIVERSITY OF PUERTO RICO, UNIVERSIDAD DE PUERTO RICO
MEDICAL SCIENCES CAMPUS, RECINTO DE CIENCIAS MÉDICAS
SCHOOL OF MEDICINE, ESCUELA DE MEDICINA

DEPARTMENT OF ANATOMY AND NEUROBIOLOGY
DEPARTAMENTO DE ANATOMÍA Y NEUROBIOLOGÍA

Irene Söderhäll
Academic Editor
PLOS ONE

March 19, 2021

Dear Academic Editor, Dr. Söderhäll

Subject: Submission of revised manuscript PONE-D-21-00863

Thank you for your email on February 4, 2021 enclosing the reviewer's very helpful comments. We have thoroughly evaluated the comments and revised the manuscript accordingly. We hope this new version is now suitable for publication. Please, find below our point-by-point responses and we look forward to hearing from you soon.

Sincerely,

María A. Sosa, PhD
Professor & Chair
Dept. of Anatomy & Neurobiology
School of Medicine UPR MSC

Responses to Reviewer #1: *(page and line references in parenthesis refer to the "Manuscript with Track Changes" version)*

1. ***Reviewer:*** *The title seems long and cumbersome. I suggest perhaps "De novo assembly of the freshwater prawn Macrobrachium carcinus brain transcriptome for identification of potential targets for antibody development". I think the rest of the original title is actually implicit ---if antibodies are being generated against M. carcinus sequences from brain, these antibodies will by definition be useful as markers in the nervous system*.

   Response: When writing the original title, we wanted to be very descriptive to give the reader the fairest idea of what the content of the manuscript would be. However, we agree that the title suggested by reviewer #1 is shorter and at the same time descriptive and reads more clearly. We have thus changed it to: "*De novo* assembly of the freshwater prawn *Macrobrachium carcinus* brain transcriptome for identification of potential targets for antibody development" (page 1, lines 2-3).

2. **Reviewer:** *Although glutamate is often referred to as an "excitatory neurotransmitter", this is actually a misnomer because the effect of a transmitter is defined by the receptor ---not the transmitter. As one example provided in this manuscript, the GluR4 receptor has some inhibitory effects. I suggest rewording "excitatory neurotransmitter" (found on lines 101 and 394), e.g., generally involved in excitatory influences.*

   Response: We changed the phrase "*being one of the main excitatory neurotransmitters*" with what was suggested by the reviewer "*being generally involved in excitatory influences*" (page 4, line 100-101). We agree and understand that the excitatory response falls on the receptor rather than the neurotransmitter. The sentence in page 17 lines 407-409 was also modified to make this concept clear, eliminating "*as one of the most abundant neurotransmitters*" and "*and the main neurotransmitter*" to include the phrase "*generally eliciting excitatory responses*".

3. **Reviewer:** *I believe that the models in part D of Figures 3-7 actually describe the tertiary structure of these contig sequences (not secondary structure as stated in the text and legends), because folds indicative of the side chain interactions are indicated.*

   Response: This is very true. After revising the figures and looking at the information provided, the models shown in part D of Figures 3-7 represent the compact 3D structure of the tertiary folding caused by the interactions of the different protein domains. Thus, we have replaced the word "secondary" in the text and figure captions with the word "*tertiary*" (page 11, line 266; page 12, lines 277 and 293; page 13, lines 303 and 320; page 14, lines 330 and 344; page 15, lines 355, 370, and 380).

4. We accept all the minor editorial suggestions made by reviewer #1:

   a. ***Line 54: constituents "of" (not to)***
      We replaced "to" with "of" (page 3, line 54).
   b. ***Line 86: American lobster ("American" should be capitalized)***
      "american" was capitalized - "American lobster" (page 4, line 86)
   c. ***Line 92: "…and pigment-dispersing hormone" ("the" is awkward here)***
      "the" was eliminated (page 4, line 92).
   d. ***Lines 105-108: This sentence is long and difficult to read.***
      This sentence was reworded as follows: "Feinstein and colleagues (26) showed NMDA antibody staining at the presynaptic membranes of neuromuscular junctions in the crayfish *Procambarus clarkii."* (page 5, lines 106-109).
   e. ***Line 110: "specific in the thoracic appendages" (not "at")***
      "at" was replaced by "in" (page 5, line 112)

f. ***Line 131: "continuously filtered"***

"continuous" was replaced by "continuously" (page 6, line 136)

g. ***Line 144: "filter cartridge" does not need to be capitalized***

uppercase letters in "Filter Cartridge" were changed to lowercase (page 6, line 150)

h. ***Line 165: "related to" (rather than "with")***

"related with" was replaced by "related to" (page 7, line 172)

i. ***Line 187: "…markers in the prawn CNS and neuromuscular junction…"***

The sentence was reorganized, "prawn" was placed before CNS (page 8, line 194).

j. ***Line 211: "a total…was annotated"***

"were" was replaced by "was" (page 9, line 219)

k. ***Line 220: "associated with" (not "to")***

"with" was used instead of "to" (page 10, line 229)

l. ***Line 239: "which contigs" (not "what")***

"what" was replaced by "which" (page 10, line 249)

m. ***Line 251: "when examined individually" (not "looked")***

"looked" was replaced by "examined" (page 11, line 261)

n. ***Line 252: "above 1, are predicted to elicit"***

deleted "going" and inserted "predicted" (page 11, line 262)

o. ***Line 265: I suggest that after the comment "showing 6 determinants", you might include in parenthesis "(peaks in graph)" for those who are not familiar with these plots.***

The parenthesis was inserted after "showing 6 determinants" (page 11, line 276) and also was inserted in all figure's caption (page 12, line 302; page 14, line 329 and 354; page 15, line 379)

p. ***Line 270: "shows reliable sequence conservation" (remove "a")***

"a" was eliminated from the sentence (page 12, line 281)

q. ***Line 341: "European" and "Indian" should be capitalized.***

"European" and "Indian" were capitalized as proper nouns (page 13, line 310 and 327; page 14, line 352)

r. ***Line 379: use present tense here? "…this is the first study that reports genomic information"***

Substituted "will" with "reports" (page 16, line 392)

s. ***Line 392: "on identifying" (rather than "in")***

"in identifying" was replaced by "on identifying" (page 16, line 405)

t. ***Line 397: "…about glutamate receptor distribution and localization…" (possessive not necessary)***
"about its receptor's distribution…" was replaced with "about glutamate receptor distribution…" (page 17, line 410)

u. ***Line 406: "Xenopus laevis"??***
added the complete species name "*Xenopus laevis*" (page 17, line 419)

v. ***Line 430: "…and their most potent agonist…" (should be plural, "their")***
"it most potent…" was replaced by "their most potent" to make it plural (page 18, line 443)

w. ***Line 438: This needs clarification, as it's not clear what a "mammalian-raised" antibody is. I believe you are trying to say "…using an antibody raised against the mammalian form of the receptor…"***
This is indeed what we actually wanted to say. The sentence was modified as suggested. (page 18, lines 451 and 452)

x. ***Line 440: "This provides the advantage…"***
"gives" was replaced by "provides" (page 18, line 454)

y. ***Line 443: "increasing the reproducibility of labeling."***
added "of labeling" after "…increasing the reproducibility" (page 18, line 456)

z. ***Line 444: "With the transcriptome contribution and the availability…"***
The beginning of the sentences was reworded to: "With the transcriptome contribution and the availability…" (page 19, line 463)

Responses to Reviewer #2: *(page and line references in parenthesis refer to the "Manuscript with Track Changes" version)*

1. **Methods: Need more detail about tissue used in generating the transcriptome.**
   a. **Was only one transcriptome generated? (Why not more than one, for statistical purposes?)**
   Only one transcriptome was generated, as part of a graduate course, and for use to complement an aim of a graduate student´s dissertation project. Our objective was to obtain transcript sequences, at normal physiological conditions, related to ionotropic and metabotropic glutamate receptors to use them as reference for antibody design. To our understanding, more than one transcriptome would be needed when, for example, assessing differential gene expression between two or more conditions (Li,

2019), where statistical analyses would be required. These types of experiments are being planned for the near future, but we believe it is important to make the present transcriptome available to others now.

Li D. Statistical Methods for RNA Sequencing Data Analysis. In: Husi H, editor. Computational Biology [Internet]. Brisbane (AU): Codon Publications; 2019 Nov 21. Chapter 6. Available from: https://www.ncbi.nlm.nih.gov/books/NBK550334/ doi:10.15586/computationalbiology.2019.ch6

b. How many animals were used?

A total of four (4) biological samples were used in generating the transcriptome (page 5, line 132).

c. Why use males only? (what might be the consequences of this with respect to capturing of the glutamate receptors of interest?)

Only males were used because of requirements of the collecting permits issued by the Department of Environmental and Natural Resources, that seek to avoid perturbation of the female´s egg release and eclosion cycles in the rivers of Puerto Rico. While there is evidence of sex differences in glutamate receptor gene expression profiles (upregulation or downregulation) in mice and other species, to our knowledge there have been no reports of glutamate receptor types that are expressed *solely* in one of the sexes. Less is known about sex differences in glutamate receptor expression in crustaceans. For instance, it has been reported that male sex differentiation in decapod and cladoceran crustaceans is mediated by an activation of ionotropic glutamate receptors, especially NMDA subtypes (Toyota et al., 2021, 2015). Nevertheless, since the focus of the present study is to develop markers of crustacean neuromuscular junction synaptic morphology and structure, glutamate receptors identified through a male prawn transcriptome will be adequate, at least for assessing these parameters in male prawns. We will be able to expand on this knowledge and better study potential sex differences in glutamate receptor expression in future experiments where female *M. carcinus* prawns become more readily available for research, for example by raising in the lab or obtaining through aquaculture farms.

Toyota, K., Miyakawa, H., Hiruta, C., Sato, T., Katayama, H., Ohira, T., & Iguchi, T. (2021). Sex determination and differentiation in decapod and cladoceran crustaceans: An overview of endocrine regulation. Genes, 12(2), 1–16. https://doi.org/10.3390/genes12020305

Toyota, K., Miyakawa, H., Yamaguchi, K., Shigenobu, S., Ogino, Y., Tatarazako, N., … Iguchi, T. (2015). NMDA receptor activation upstream of methyl farnesoate signaling for short day-induced male offspring production in the water flea, Daphnia pulex. BMC Genomics, 16(1), 186.

https://doi.org/10.1186/s12864-015-1392-9

d. **What about conditions of the animals used? (size and weight? molt stage? how long were they in the lab after capture in the wild before being used)?**

Animal	Size (cm)	Weight (g)
1	14.0	74.70
2	13.3	59.52
3	11.3	41.31
4	13.1	61.88

Animals were not categorized or selected by molt stages. Once the animals arrived at the laboratory facility, they spent ten (10) days in the conditions stated in the methodology until dissection and total RNA extraction (clarification of this has been added, page 6, line 137).

e. **It is stated that "brain ganglia" were collected, but what is meant by this? Supraesophageal ganglion? No subesophageal ganglion? Eyestalks with all ganglion and retina? All nerve roots including circumesophageal connectives?**
Only the supraesophageal ganglion of the ventral nerve cord was isolated for total RNA extraction (clarification of this has been added, page 6, line 146)

2. **Give more information about the intent of the study, especially about the desired specificity of the antibodies and whether the methods have achieved that.**

The objective of this study is to develop a de novo transcriptome from the brain (supraesophageal ganglia) of the freshwater prawn *M. carcinus* to design specific antibodies against glutamate receptors in the crustacean. Our main goal was to generate antibodies that work well in crustacean tissue, since our trials with commercially available antibodies raised in other species have resulted in high non-specific binding and very low consistency of immunohistochemistry staining profiles in *Macrobrachium carcinus* prawns, the most abundant local species of prawn. Our lab uses this species to assess the impact of environmental perturbations, such as exposure to emerging contaminants or temperature variations, on nervous system structure and function. It is with this intent of finding antibodies better suited for *M. carcinus* tissue that we decided to explore sequences obtained from a species-specific transcriptome.

The purpose of performing multiple sequence alignments of the glutamate receptor sequences identified in the prawn transcriptome through comparisons with different

species (crustacean, insects, human, monkeys, etc.) was to look for those fragments within the sequences with high degree of conservation, suggesting important functional domains for evaluation as potential strong antigenic targets for antibody recognition. With this we are seeking to improve the specificity of the antibodies for recognizing glutamate receptors and not other proteins in the prawn tissue, as has been the case with mammalian-based commercially available antibodies. We understand this point was not clear in the text of our manuscript, as it seemed to imply that we are seeking species specificity rather than receptor specificity. We have thus reworded the original text for better clarity about these points, as follows:

Page 5, lines 116-118 – added the sentence "Unfortunately, in our hands some of the commercially-available glutamate receptor antibodies used with nervous system tissue of Macrobrachium prawns appear to show non-specific binding."

page 5, lines 120-121 – reworded "specific antibodies" to "antibodies specific to glutamate receptors"

page 5, line 126 – reworded "…develop crustacean nervous system markers" to "…develop nervous system markers that work well in Macrobrachium species…"

Page 10, line 244 – added "…suggesting likely important functional domains,…"

Page 18, lines 456 to 458 – reworded "…decreasing the chances of experiencing non-specific binding with other progeins" to "…increasing the reproducibility of labeling and decreasing the chances of experiencing non-specific binding with other crustacean proteins, which had been our greatest difficulties with commercially available glutamate receptor antibodies."

Pages 18-19, lines 458 to 462 – added the suggested two sentences: **"**Moreover, the results presented here are important because not only are they useful for generating antibodies, but also for generating nucleotide sequences as probes for in situ hybridization experiments. This offers a very efficient method to describe changing expression patterns of receptors of interest due to environmental influences. "

3. **Data availability:** Crustybase does not accept transcriptomes that do not include information on differential expression or at least two experimental conditions for comparison. We have instead submitted and registered our transcriptome files and the glutamate receptors sequences with the National Center for Biotechnology Information (NCBI) GenBank, through their tool "Transcriptome Shotgun Assembly Sequence Database" (TSA). We have already

created a BioProject (Accession Number: PRJNA716066) for the submission (ID: SUB9330334). Once the NCBI staff completes the required review and provides us with the accession number of the transcriptome file, we will provide it. The link to the transcriptome, presently set as http://www.ncbi.nlm.nih.gov/bioproject/716066, has been added to the manuscript, in page 9, line 216, in addition to the reference to Supplement File 1 (S1 File). The date for release of the database has been preliminarily set for May 21, 2021, and can be modified as needed, to allow time for coordinating the release with the publication date for the manuscript.

4. We accept all the minor editorial suggestions made by reviewer #2:
   a. ***Line 56: "destroying" is not the correct word***
      replaced the word "destroying" with "consuming" (page 3, line 56)
   b. ***Line 63: This reads "The depth of the studies characteristic specificity….is a current research focus." This does not make sense. Perhaps delete "The depth of the studies"?***
      deleted "The depth of studies characterizing specific…" and replaced it with "Characterization of…" (page 3, line 63)
   c. ***Line 75: "SNPs" not "SNP's"***
      The apostrophe was deleted (page 3, line 75)
   d. ***Line 86: "American" not "american"***
      "American" was capitalized (page 4, line 86)
   e. ***Line 110: should be spelled "amphitrite"***
      "y" was replaced by "i" in *amphitrite* (page 5, line 112)
   f. ***Line 123: "spp." should not be italicized***
      "spp" is not italicized anymore (page 5, line 126)
   g. ***Daphnia is called a "microcrustacean." It is small, but what is most relevant is its phylogeny relative to the other species. The authors should use modern phylogenetic terms – perhaps using Schwentner et al. 2017 Current Biology or some other scheme. Daphnia should be called a cladoceran or branchiopod.***
      The word "microcrustacean" was replaced by "branchiopod" (page 11, line 254 and 273; page 18, line 439)
   h. ***Line 316: "Indian jumping ant" (capital "I")***
      "Indian" is capitalized (page 13, line 311 and 327; page 14, line 352)
